# A Substrate-Activated Efflux Pump, DesABC, Confers Zeamine Resistance to Dickeya zeae

**DOI:** 10.1128/mBio.00713-19

**Published:** 2019-05-28

**Authors:** Zhibin Liang, Luhao Huang, Fei He, Xiaofan Zhou, Zurong Shi, Jianuan Zhou, Yufan Chen, Mingfa Lv, Yumei Chen, Lian-Hui Zhang

**Affiliations:** a Guangdong Province Key Laboratory of Microbial Signals and Disease Control, South China Agricultural University, Guangzhou, China; b Integrative Microbiology Research Centre, South China Agricultural University, Guangzhou, China; National Cancer Institute; University of Cambridge; International Centre for Genetic Engineering and Biotechnology

**Keywords:** RND efflux pump, antimicrobial resistance, phytotoxin, rice stem rot, zeamines

## Abstract

Zeamines are a family of polyamino phytotoxins produced by Dickeya zeae EC1. These phytotoxins are also potent antibiotics against a range of microorganisms. To understand how *D. zeae* EC1 can protect itself from the antimicrobial activity of zeamines, we tested whether the ABC transporter genes within the *zms* (zeamine synthesis) gene cluster were related to zeamine resistance. Our results ruled out the possible involvement of these ABC transporters in zeamine resistance and instead unveiled an RND (resistance-nodulation-cell division) efflux pump, DesABC, which plays an important role in zeamine resistance in *D. zeae* EC1. The *desAB* genes are located next to the *zms* gene cluster, but *desC* is at a distant location in the bacterial genome. Null mutation of the *desABC* genes in a zeamine-minus derivative of strain EC1 led to about an 8- to 32-fold decrease in zeamine tolerance level. This efflux pump was zeamine specific and appeared to be conserved only in *Dickeya* species, which may explain the high potency of zeamines against a wide range of bacterial pathogens. Significantly, expression of the *desAB* genes was abolished by deletion of *zmsA*, which encodes zeamine biosynthesis but could be induced by exogenous addition of zeamines. The results suggest that sophisticated and coordinated regulatory mechanisms have evolved to govern zeamine production and tolerance. Taken together, these findings documented a novel signaling role of zeamines and the first resistance mechanism against zeamines, which is a family of potent and promising antibiotics against both Gram-positive and Gram-negative bacterial pathogens.

## Abstract (precis)

Zeamines are a family of newly identified phytotoxins and potent antibiotics produced by *D. zeae* EC1. Unlike most bacterial organisms, which are highly sensitive, *D. zeae* EC1 is tolerant to zeamines, but the mechanisms involved are unknown. Our study showed, for the first time, that a new RND efflux pump, DesABC, is indispensable for *D. zeae* EC1 against zeamines. We found that the DesABC efflux pump was zeamine specific and appeared to be conserved only in the *Dickeya* species, which may explain the high potency of zeamines against a wide range of bacterial pathogens. We also showed that expression of DesABC efflux system genes was induced by zeamines. These findings not only provide an answer to why *D. zeae* EC1 is much more tolerant to zeamines than other bacterial pathogens but also document a signaling role of zeamines in modulation of gene expression.

## INTRODUCTION

The phytopathogen Dickeya zeae can cause severe infections on both dicotyledonous and monocotyledonous plants (1). Similar to other species in the *Dickeya* genus, the virulence of *D. zeae* is linked to cell motility, biofilm formation, and production of cell wall-degrading enzymes (1–3), except that *D. zeae* also produces a family of phytotoxins, known as zeamines (4, 5). Zeamines are a family of structurally related polyamino compounds that play important roles in the pathogenicity of *D. zeae* EC1. Inactivation of *zmsA*, the key gene responsible for the biosynthesis of all zeamine compounds, abrogated the infectivity of *D. zeae* EC1 on rice, potato, and Chinese cabbage (4).

Zeamines are produced by *Dickeya* species and Serratia plymuthica strains with the *zms* gene cluster, including *D. zeae* EC1 and *S. plymuthica* RVH1 (4–9). Among them, zeamine, zeamine I, and prezeamines are the derivatives of a polyamino chain zeamine II, with polyketide moiety conjugating at the terminal amino group of zeamine II (4–7, 9). Apart from their important role in the virulence of *D. zeae* EC1, zeamines are also potent antibiotics with broad-spectrum activity against various organisms, including multidrug-resistant bacteria, fungi, oomycetes, and nematodes (8–10). Evidence shows that zeamines target the outer membrane of Gram-negative bacteria in a way reminiscent of the cationic antimicrobial peptide polymyxin B (11). Organization of the *zms* gene clusters is genetically well conserved in *D. zeae* EC1 and *S. plymuthica* RVH1, with genes encoding polyketide synthases (PKSs), nonribosomal peptide synthetases (NRPSs), and fatty acid synthases (FASs). In addition, five genes within the *zms* gene cluster were predicted to encode transporter proteins, including four encoding putative ATPases and permeases associated with the ABC transporter system and one encoding a potential HlyD superfamily protein (6, 8, 12). One of the predicted ABC transport systems encoded by *zmn20* and *zmn21* was proposed to be a zeamine transporter and associated with zeamine resistance in *S. plymuthica* RVH1 (6), but this speculation has not yet been validated experimentally.

Multidrug resistance (MDR) efflux pumps are membrane-associated proteins that can export a wide range of antibiotics and confer intrinsic antibiotic-resistant ability to bacteria. The efflux pumps can be classified into five superfamilies: MFS (major facilitator superfamily), ABC (ATP-binding cassette), SMR (small multidrug resistance), MATE (multidrug and toxic compound extrusion), and RND (resistance-nodulation-cell division) (13). In Gram-negative bacteria, RND efflux pumps play important roles in MDR due to their broad-spectrum substrate profile (14). The RND efflux pump is a tripartite complex system comprised of an outer membrane channel, an adaptor, and an inner membrane protein, all of which are required for the full function of antibiotic transportation (15). The genes responsible for encoding RND efflux pumps are commonly presented as a single operon in bacteria, like the MexAB-OprM efflux pump in Pseudomonas aeruginosa (16), but there are also exceptional cases with the gene encoding outer membrane channel protein placed in another location in the genome (17). In RND efflux pumps, antibiotic specificity is determined by the inner membrane protein. Antibiotics belonging to different families can enter into the inner membrane proteins through three putative entrance channels opening to the central cavity of inner membrane protein, the inner membrane, and periplasmic space of bacterial cells (18). The multiple active binding sites in the porter region of inner membrane proteins make it possible for the RND efflux pumps to transport a variety of structurally unrelated antibiotics produced by bacteria themselves (19) or from the environment (20, 21).

How *D. zeae* EC1 protects itself from the antimicrobial activity of zeamines remains unknown. While the MICs of zeamines for most bacterial pathogens are low, in the range of 0.3 to 10 μg/ml (9), our preliminary assay results showed that the zeamine producer *D. zeae* EC1 could tolerate up to 1,800 μg/ml of zeamines, suggesting a high-level resistance mechanism(s) is encoded by the *D. zeae* EC1 genome. In this study, we tested whether the transporter genes within the *zms* gene cluster, which were speculated to play roles in zeamine resistance (6), and the adjacent genes encoding RND efflux pump are associated with zeamine resistance in *D. zeae* EC1. Our results rule out the possible involvement of the ABC transporter genes within the *zms* gene cluster in zeamine resistance but lead to identification of a RND efflux pump, DesABC, that confers a high level of zeamine tolerance in *D. zeae* EC1. Substrate specificity assay against a range of antibiotics showed that DesABC appeared to only confer resistance against zeamines. In addition, the DesABC efflux system was found to be functionally conserved in *Dickeya* species. Interestingly, consistent with the zeamine-specific pattern of DesABC, we found that the transcriptional expression of its coding genes, *desAB*, was stimulated by the presence of zeamines, suggesting that DesABC co-evolved with the genes encoding zeamine biosynthesis to ensure high-level production of the antibiotics.

## RESULTS

### The ABC transporter systems encoded by the *zms* gene cluster are not required for zeamine resistance in *D. zeae* EC1.

**FIG 1:**
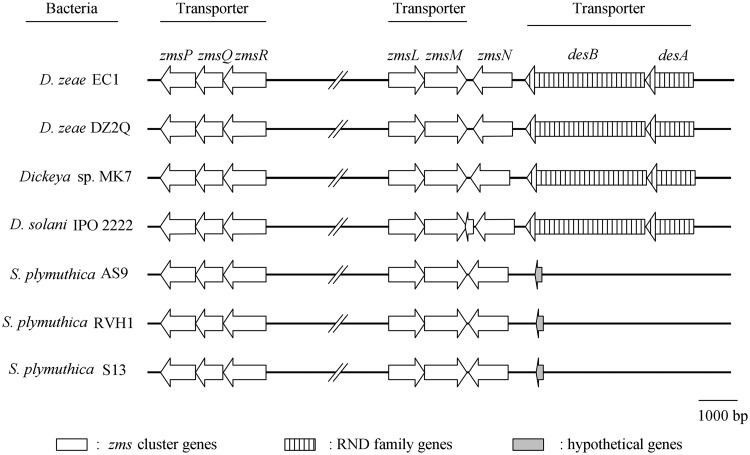
Organization of transporter genes within and adjacent to the *zms* gene cluster in *Dickeya* species and Serratia plymuthica strains. The organization of genes was drawn using Illustrator for Biological Sequences (34). Data were derived from NCBI and updated to 24 July 2018.

As the transporter systems present within the antibiotic biosynthesis gene clusters often confer resistance to the encoded antibiotics, we conducted bioinformatics analysis of the five transporter genes, i.e., *zmsP*, *zmsQ*, *zmsR*, *zmsL*, and *zmsM* (NCBI accession no. WP_016943528.1, WP_016943529.1, WP_016943530.1, WP_016943542.1, and WP_016943543.1, respectively) within the *zms* gene cluster of *D. zeae* EC1 (Fig. 1) (12). These genes share similar genetic organization (Fig. 1) and high levels of identity and similarity in amino acids (above 69% and 82%, respectively) (Table S2) to their homologs found in *S. plymuthica* and other *Dickeya* species and strains with the *zms* gene clusters (8). Among them, sequence analysis showed that *zmsP* encodes a potential HlyD family protein, *zmsR* and *zmsM* encode potential ABC transporter permeases, and *zmsQ* and *zmsL* encode potential ABC transporter ATPases. The homologs of *zmsM* and *zmsL* were hypothesized to encode a zeamine transporter, conferring self-resistance against zeamines in *S. plymuthica* RVH1 (6). As a previous study indicated that the HlyD family protein could form a functional transport system with ABC transporter proteins (22), ZmsP was considered a part of the ABC transporter systems encoded by the *zms* gene cluster. To elucidate the potential roles of these genes in zeamine resistance, in-frame deletion was performed to generate the deletion mutants of *zmsR* and *zmsM*, respectively, which represent the two putative transport systems encoded by the *zms* gene cluster of *D. zeae* EC1. Zeamines were purified from the cell culture of *D. zeae* EC1 and confirmed by liquid chromatography-mass spectrometry (LC-MS) (see Fig. S1 in the supplemental material) and used for determination of MIC against different bacterial strains in this study. The results showed that inactivation of *zmsR* or *zmsM* could not cause any change in the MIC of zeamines compared with those of the wild-type strain EC1 (data not shown). These findings preclude the possible association of these ABC transport systems with zeamine resistance, and their roles remain to be further investigated.

10.1128/mBio.00713-19.1 FIG S1 High-resolution mass spectrum of zeamine II (A), zeamine I (B), and zeamine (C) detected by LC-MS in purified zeamine samples. The structure was drawn by using the software ChemBioDraw Ultra 14.0 (Cambridgesoft). Download FIG S1, PDF file, 0.3 MB. Copyright © 2019 Liang et al. 2019 Liang et al. This content is distributed under the terms of the Creative Commons Attribution 4.0 International license.

10.1128/mBio.00713-19.6 TABLE S2 Identity and similarity of the transporter genes within and adjacent to the *zms* gene clusters in *Dickeya* species and Serratia plymuthica strains. Download Table S2, DOCX file, 0.03 MB. Copyright © 2019 Liang et al. 2019 Liang et al. This content is distributed under the terms of the Creative Commons Attribution 4.0 International license.

### DesABC efflux system is required for zeamine resistance.

In addition to the five potential transporter genes within the *zms* gene cluster, further bioinformatics analysis unveiled two genes encoding RND efflux pump proteins located adjacent to the *zms* gene cluster of *D. zeae* EC1 and another three *Dickeya* species and strains (Fig. 1). One of the gene clusters encodes a putative AcrA-like adaptor protein, and the other encodes a potential AcrB-like inner membrane protein. The AcrAB-TolC RND efflux system has been well characterized as being associated with multiple antibiotic resistance in Escherichia coli, including β-lactams, tetracycline, chloramphenicol, and rifampin, with *acrAB* located together in the genome and *tolC* at a distant location (15). We proposed to name these two genes *desA* and *desB*, for *Dickeya* efflux system proteins A and B (Fig. 1). Interestingly, the *desAB* genes are not present within the vicinity of the *zms* gene cluster in *S. plymuthica* strains (Fig. 1).

Topological analysis of DesB revealed typical features of inner membrane transporter protein in an RND efflux system with 12 transmembrane helix domains (TM) and 2 large periplasmic loops spanning from TM1 to TM2 and TM7 to TM8 (Fig. S2) (23). In RND efflux systems, a tripartite complex is required for the full function of substrate transportation. To identify the outer channel protein for DesAB, a BLAST search was conducted to identify the homolog of E. coli outer membrane protein TolC. The result showed that only one *tolC* homolog (74% identity and 86% similarity at the amino acid level) is present in the genome of *D. zeae* EC1, which was designated *desC* accordingly. To elucidate the role of the DesABC system in zeamine resistance, three *des* genes were deleted in-frame separately at the background of the strain defective in zeamine production, i.e., *zmsA* in-frame deletion mutant. Inactivation of *desA* and *desB* led to about an 8-fold decrease in the MIC of zeamines, while deletion of *desC* led to about a 32-fold decrease in MIC (Table 1). Consistent with the above-described results, in *trans* expression of *desB* and *desC* in the corresponding mutants could increase the zeamine resistance level of the mutants (Table 1). In addition, we found that heterologous expression of *desABC* under the control of the *lac* and *tetO* promoter in E. coli DH5α increased the MIC of zeamines by 2-fold (Table 1). These results demonstrate the role of the RND system DesABC in self-protection of *D. zeae* EC1 against the antimicrobial activity of zeamines.

**TABLE 1:** Zeamine susceptibility of Dickeya zeae and Escherichia coli derivatives

Strain	ZEA^*a*^ MIC (μg/ml)
Δ*zmsA*	1,800
Δ*zmsA*Δ*desB*	225
Δ*zmsA*Δ*desA*	225
Δ*zmsA*Δ*desC*	56.25
Δ*zmsA*Δ*desB*(pBB-*desB*)	1,800
Δ*zmsA*Δ*desB*(pBB-*desB_3937_*)	1,800
Δ*zmsA*Δ*desC*(pBB-*desC*)	900
DH5α	3.52
DH5α(pBB, pAmob)	3.52
DH5α(pBB-*desAB*, pAmob-*desC*)	7.03

a ZEA, zeamines.

10.1128/mBio.00713-19.2 FIG S2 Predicted topological structure of DesB in cell membrane. TMHMM (http://www.cbs.dtu.dk/services/TMHMM/) was used to analyze DesB amino acids, and the result was illustrated using TMRPRES2D software. Download FIG S2, PDF file, 0.3 MB. Copyright © 2019 Liang et al. 2019 Liang et al. This content is distributed under the terms of the Creative Commons Attribution 4.0 International license.

### DesABC efflux system is zeamine specific and functionally conserved in *Dickeya* species.

DesABC belongs to the RND efflux systems, in which the inner membrane proteins associated with recognition and binding have been well characterized to aid in understanding their substrate profiles (14). For example, MexY from P. aeruginosa is required for streptomycin resistance (24), MexB and AcrB from P. aeruginosa and E. coli are associated with chloramphenicol and tetracycline resistance (25, 26), CmeB from Campylobacter jejuni plays a role in resistance against ampicillin, chloramphenicol, gentamicin, and tetracycline (27), and AdeB in Acinetobacter baumannii BM4454 is involved in tetracycline, chloramphenicol, gentamicin, and kanamycin resistance (28). To understand the potential substrate profile of the DesABC efflux pump, a phylogenic tree was constructed with DesB of *D. zeae* EC1 and its homologs (sequence similarity above 93%) found by blastp search in *Dickeya* species, other proteobacterial species, including the homologs (sequence similarity above 80%) from *S. plymuthica* strains containing the *zms* gene cluster, as well as the above-mentioned inner membrane proteins with known functions (Table S3). The DesB phylogeny was largely consistent with known evolutionary relationships among the bacterial genomes. All of the *Dickeya* DesB proteins formed a monophyletic clade in the tree, whereas the homologs from other genera were more distantly related (Fig. S3). Notably, the inner membrane proteins with known substrate profiles were clustered together on the tree and showed considerable divergence from the DesB homologs from *Dickeya* species. The result suggests that DesB and its homologs from *Dickeya* species have a different substrate profile than their counterparts from other bacterial species.

10.1128/mBio.00713-19.3 FIG S3 Phylogenic relationship of DesB with the inner membrane proteins of other RND efflux pumps. The black dots show bootstrap support higher than 95%. The underlines show the positions of DesB from Dickeya zeae EC1 and DesB_3937_ from Dickeya dadantii 3937. The black triangles show the proteins whose substrate profiles were determined previously. Download FIG S3, DOCX file, 1.8 MB. Copyright © 2019 Liang et al. 2019 Liang et al. This content is distributed under the terms of the Creative Commons Attribution 4.0 International license.

10.1128/mBio.00713-19.7 TABLE S3 Characteristics of RND family genes used in phylogenic study. Download Table S3, DOCX file, 0.03 MB. Copyright © 2019 Liang et al. 2019 Liang et al. This content is distributed under the terms of the Creative Commons Attribution 4.0 International license.

The substrate profile of DesABC was then examined experimentally by MIC assay. The results showed that in *trans* expression of *desB* from *D. zeae* EC1 or *desB_3937_* from *D. dadantii* 3937, which lacks the *zms* gene cluster, in the *zmsA*-*desB* double deletion mutant of *D. zeae* EC1 could fully restore the zeamine resistance (Table 1), suggesting that the *desB* orthologs from other *Dickeya* species (Table S3) have a similar function in zeamine resistance. MIC assay was also performed using antibiotics which belong to different classes and have different targets (Table S4). The results showed that neither inactivation of *desB* nor overexpression of *desAB* genes in *D. zeae* EC1 could affect the MICs of ampicillin, tetracycline, kanamycin, gentamicin, streptomycin, and chloramphenicol (Table S5). The above data are consistent with the phylogenetic analysis results (Fig. S3), indicating that the DesABC system has a distinct substrate specificity.

10.1128/mBio.00713-19.8 TABLE S4 Classes and targets of antibiotics. Download Table S4, DOCX file, 0.02 MB. Copyright © 2019 Liang et al. 2019 Liang et al. This content is distributed under the terms of the Creative Commons Attribution 4.0 International license.

10.1128/mBio.00713-19.9 TABLE S5 Antibiotic susceptibility of the *desB* mutant, *desAB* overexpression strain, and their parental strains. Download Table S5, DOCX file, 0.02 MB. Copyright © 2019 Liang et al. 2019 Liang et al. This content is distributed under the terms of the Creative Commons Attribution 4.0 International license.

### DesABC efflux system is essential for *D. zeae* survival against zeamines.

**FIG 2:**
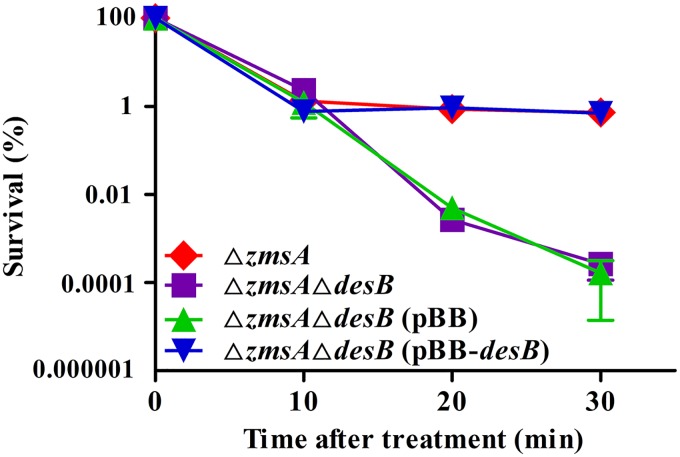
Survival analysis of the *desB* mutant and its parental and complementation strains treated with zeamines. Bacterial cells were measured at 10, 20, and 30 min after treatment with zeamines. The survival rate was expressed as the percentage of the colony counts of the control not exposed to zeamines. Data in the graph are the means from three repeats, and error bars are standard deviations.

As the DesABC system was found to be required for zeamine resistance, a survival assay was conducted against zeamines using the *zmsA* mutant, the *zmsA-desB* double deletion mutant, and the double mutant complemented with *desB*. Bacterial cells were added to LS5 salts, without carbon source, supplemented with zeamines at a final concentration of 2-fold the MIC of the *zmsA*-*desB* double deletion mutant, and bacterial cell numbers were measured at different time points upon treatment with zeamines to evaluate the role of DesABC in *D. zeae* EC1 survival. The results showed that inactivation of *desB* resulted in a sharp decline in survival rate, whereas its parental and complemented strains could maintain upon to a three-log larger amount of survivors than the *desB* mutant at 30 min after treatment (Fig. 2). These results indicate that the DesABC efflux pump plays an indispensable role in the survival of strain EC1 when the bacterial cells were treated with zeamines.

### DesABC efflux system confers bacterial self-resistance against zeamines at the late stage of bacterial growth.

**FIG 3:**
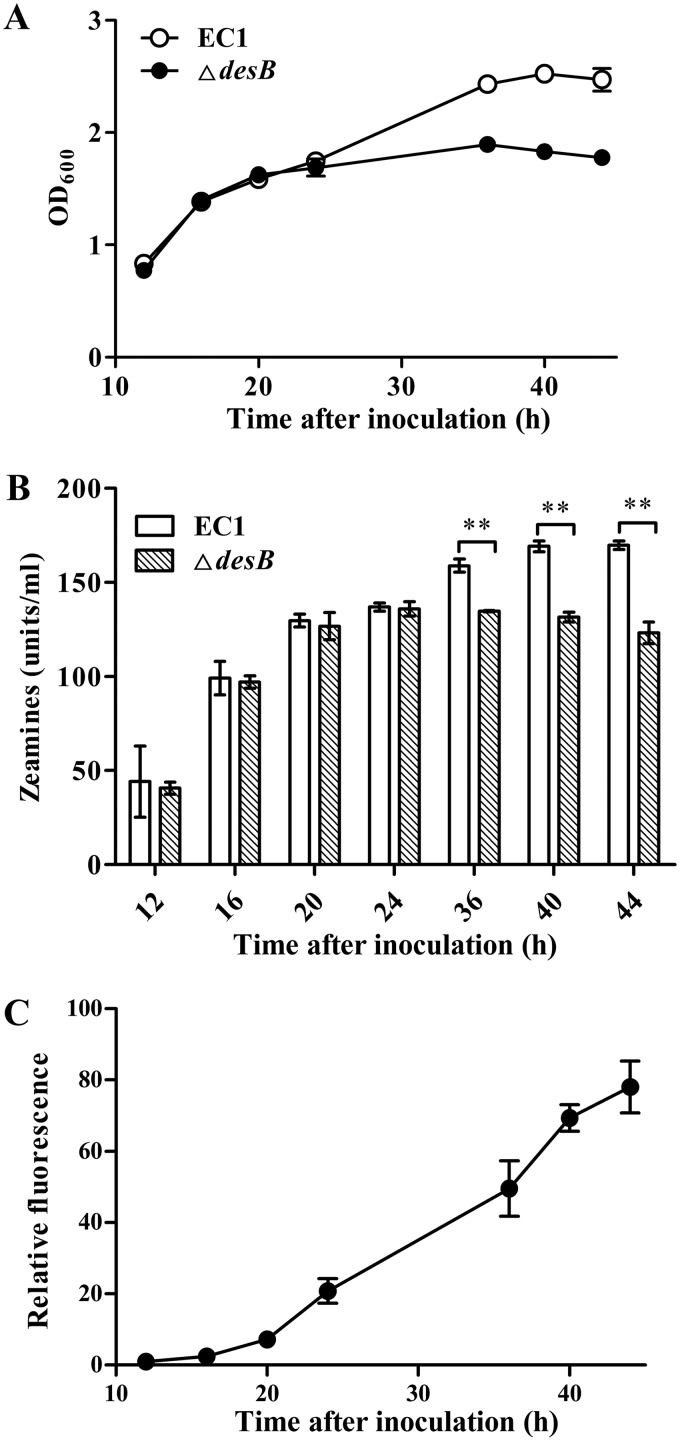
Analysis of the bacterial growth, zeamine production, and the expression pattern of *desAB*. (A) Growth kinetics of wild-type strain EC1 and its *desB* mutant. Cell cultures at different time points were collected for measuring the OD_600_ for plotting the growth curves. (B) Zeamine production profiles of strain EC1 and its *desB* mutant. The collected bacterial culture supernatants were filter sterilized for quantification of zeamine production. (C) Transcriptional fusion assay to determine the expression pattern of *desAB* in *D. zeae*. Strain EC1 containing the expression construct pDesAB_gfp_ was cultured in flasks with LS5 medium. Cell cultures at different time points were collected for monitoring the fluorescence. The relative fluorescence was expressed as the fluorescence monitored at specific time points normalized to the fluorescence of EC1(pDesAB_gfp_) at 12 h after inoculation. Data in the graph are the means from three repeats, and error bars are standard deviations. Significant values are indicated by bars and asterisks. **, *P < *0.01.

To investigate the protective spectrum of DesABC during cell growth, an in-frame deletion mutant of *desB* was constructed using *D. zeae* wild-type strain EC1. The cell growth curves and zeamine production of wild-type strain EC1 and *desB* mutant were compared in LS5 medium, which was optimized for zeamine production (29). At the early stage after inoculation (12 to 24 h), the growth rate and zeamine production were comparable between strain EC1 and the *desB* mutant (Fig. 3A). However, the growth of the *desB* mutant was arrested in the subsequent stages after 24 h (Fig. 3A), and similarly, accumulation of zeamines in bacterial supernatant was also flattened after 24 h (Fig. 3B). These results suggest that *D. zeae* cells could tolerate a certain level of zeamines in the absence of the DesABC efflux system, but along with bacterial growth and zeamine accumulation, the DesABC efflux pump becomes indispensable for protecting bacterial cells against the detrimental effect of zeamines.

To determine the relationship between expression of DesABC genes and zeamine resistance, the *gfp* coding region was placed under the promoter of *desAB*, and the transcriptional fusion construct pDesAB_gfp_ was prepared. The expression of *desAB* was evaluated by monitoring the fluorescence of wild-type strain EC1 containing the pDesAB_gfp_ construct grown in LS5 medium by using a CytoFLEX flow cytometer system. The results showed that expression of *desAB* was bacterial population density dependent, showing a basal-level expression at the early growth stage (12 to 20 h) and rapidly increased expression at 20 h onward after inoculation (Fig. 3C). As the DesABC system was critical to the bacterial growth at the late growth stage (Fig. 3A), the above results indicate that zeamine resistance mediated by the DesABC efflux system is positively related to the expression level of *desAB* genes.

### Expression of *desAB* is induced by zeamines.

**FIG 4:**
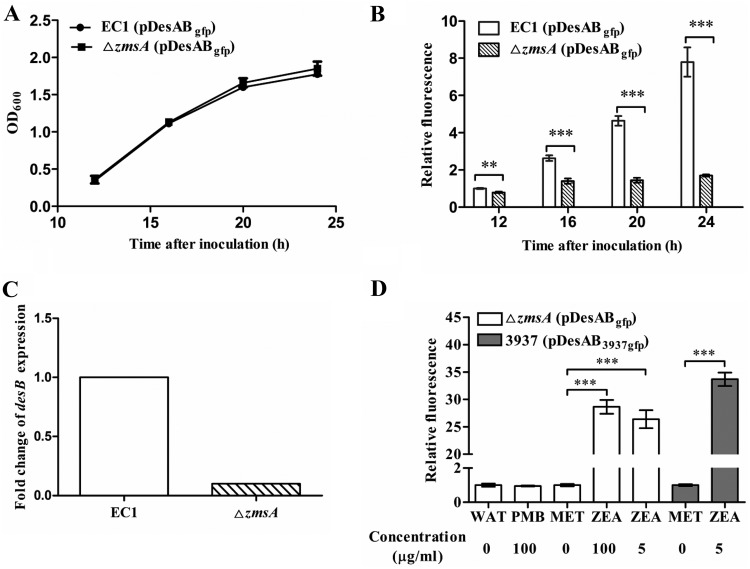
Expression of *desAB* is positively related to the exposure of zeamines. (A) Growth curves of EC1(pDesAB_gfp_) and Δ*zmsA*(pDesAB_gfp_) strains were measured in the flasks with LS5 medium at 12 h, 16 h, 20 h, and 24 h after inoculation. (B) The *desAB* expression patterns in the wild-type strain EC1 and Δ*zmsA* zeamine-minus mutant. The relative fluorescence was expressed as the fluorescence monitored at specific time points normalized to the fluorescence of strain EC1(pDesAB_gfp_) at 12 h after inoculation. (C) qPCR analysis of *desB* gene expression in strain EC1 and the Δ*zmsA* mutant. (D) Relative fluorescence of Δ*zmsA*(pDesAB_gfp_) and 3937(pDesAB_3937gfp_) strains with exogenous addition of polymyxin B (PMB) or zeamines (ZEA). The same amounts of water (WAT) and methanol (MET) were added as controls. The Δ*zmsA*(pDesAB_gfp_) or 3937(pDesAB_3937gfp_) strain was grown in LS5 medium and adjusted to an OD_600_ of about 0.5 (±0.05). Bacterial cells were then treated with different concentrations of polymyxin B or zeamines for 8 h. The relative fluorescence of cells with different treatments was normalized to the fluorescence of cells in water or methanol control, which was set as 1. Data in the graph show the means from three repeats and standard deviations (A, B, and D) or standard errors (C).

Expression of *desAB* genes was consistent with zeamine production during cell growth (Fig. 3), suggesting that *desAB* expression is influenced by zeamines. To test this possibility, the pDesAB_gfp_ construct was introduced into the *D. zeae* Δ*zmsA* mutant. Our previous results showed that deletion of *zmsA* abolished production of all the zeamines (4). The *gfp* expression level driven by the promoter of *desAB* in wild-type strain EC1 and the *zmsA* mutant was monitored at different time points during bacterial growth in LS5 medium. The results showed that the growth patterns of both strains were similar (Fig. 4A), but the expression levels of *desAB* in these strains were varied substantially (Fig. 4B). The expression of *desAB* in strain EC1 was increased along with bacterial growth but remained flat in the toxin-minus Δ*zmsA* mutant (Fig. 4B).

For validation of the findings described above, the transcript levels of the DesABC efflux system genes in *D. zeae* strain EC1 and the *zmsA* mutant were determined at an optical density at 600 nm (OD_600_) of about 1.5 (approximately 20 h after inoculation). The quantitative PCR (qPCR) results showed that although the transcript level of *desC* was comparable between strain EC1 and the Δ*zmsA* mutant (fold change of less than 2; data not shown), the *desB* transcript level in strain EC1 was significantly higher than that in the Δ*zmsA* mutant, which was hardly detectable (Fig. 4C). The basal level of expression of *desAB* noticed in the *zmsA* mutant coupled with their induced expression in wild-type strain EC1 suggest that expression of the *desAB* genes rely on the product of *zmsA*, i.e., zeamines.

To further confirm this assumption, expression of *desAB* was monitored in the Δ*zmsA*(pDesAB_gfp_) strain treated with zeamines. The result showed that a supplement of zeamines at a final concentration of 100 μg/ml did not decrease the cell growth of the Δ*zmsA*(pDesAB_gfp_) strain but led to about a 30-fold higher expression of *desAB* genes compared with that of the control without addition of zeamines (Fig. 4D). Notably, a low concentration of zeamines, 5 μg/ml, could significantly induce the expression of *desAB* genes in *D. zeae* EC1 and their homologs in *D. dadantii* 3937. As a control, we also tested whether expression of *desAB* could be induced by polymyxin B by incubation of the Δ*zmsA*(pDesAB_gfp_) strain with polymyxin B at the same concentration as zeamines. The results showed that unlike zeamines, polymyxin B could not trigger the expression of *desAB* genes (Fig. 4D). Cumulatively, these results unveil a novel and specific signaling role of zeamines in triggering the expression of the *desAB* genes in *Dickeya* species and strains.

## DISCUSSION

Unlike most bacterial pathogens, which are highly sensitive to zeamines (9, 10), the zeamine producer *D. zeae* EC1 can withstand a high level of zeamines. In this study, we identified a novel RND efflux pump, DesABC, that plays a role in resistance against zeamines, especially at the late stage of bacterial growth, when zeamines were accumulated at a high level. Null mutation of the efflux pump could lead to up to about a 32-fold decrease in zeamine resistance. In contrast, the DesABC efflux pump was not functional against a range of conventional antibiotics, including ampicillin, tetracycline, kanamycin, gentamicin, streptomycin, and chloramphenicol. Furthermore, we also showed that *desAB* expression was growth and zeamine dependent and documented a novel signaling role of zeamines in regulation of *desAB* transcription. Moreover, we found that deletion of the *desB* gene could substantially reduce the accumulation of zeamines, suggesting that zeamine biosynthesis and resistance are modulated by coordinated and sophisticated regulatory mechanisms.

The zeamine biosynthesis genes and *desAB* were clustered in the genomes of *D. zeae* EC1 and another three *Dickeya* species and strains according to bioinformatics analysis (Fig. 1). However, despite their functional relevance (Fig. 4), the results from this study suggest that *desAB* and zeamine biosynthesis genes are not tightly linked during evolution. This is evident as the genomes of multiple *Dickeya* species and strains, such as *D. zeae* Ech586 and *D. dadantii* 3937, contain *desAB* but not the zeamine biosynthesis genes. It is possible that the common ancestor of *Dickeya* contains both *desAB* and zeamine biosynthesis genes, but some *Dickeya* species and strains subsequently lost the ability to produce zeamines while maintaining DesABC as a defense mechanism. How the genes responsible for zeamine biosynthesis and resistance have originated during evolution remains to be further investigated. Interestingly, the *desAB* genes were not found in the vicinity of the *zms* gene cluster in *S. plymuthica* strains (Fig. 1), which is also known for production of zeamines (6, 7), suggesting that *D. zeae* and *S. plymuthica* have different evolutionary origins of the genes encoding zeamine biosynthesis and resistance.

The RND family proteins associated with antibiotic resistance can commonly transport a broad spectrum of substrates, which is determined by the corresponding inner membrane proteins of the efflux pumps (14). Most inner membrane proteins in the RND family found in human bacterial pathogens, including E. coli (15), P. aeruginosa (16), *Burkholderia* (30), and Acinetobacter (31), are related to transportation of multiple structurally dissimilar antibiotics. In contrast, we found that the DesABC efflux system of *D. zeae* EC1 was zeamine specific (Table 1; see also Table S5 in the supplemental material). Interestingly, two RND efflux pump systems found in Erwinia amylovora, a plant bacterial pathogen causing fire blight disease, also displayed narrow substrate specificity. Overexpression of these two RND efflux systems, MdtABC and MdtUVW, only resulted in the increment of MIC of several phytochemicals but had no effect on various conventional antibiotics (21), including some antibiotics used in our study (Table S5). Given that both *D. zeae* and E. amylovora are plant pathogens with little exposure to conventional antibiotics, these findings agree with the previous findings about the linkage between intrinsic resistance and the habitat of environmental bacteria (32). The narrow substrate specificity of MdtABC and MdtUVW in E. amylovora and DesABC in *D. zeae* EC1 may reflect the ancient role of the corresponding RND efflux pumps for bacteria to survive in hazardous environments generated by bacteria themselves or by other organisms.

Intriguingly, our results showed that inactivation of the *desABC* genes could cause different levels of decrement in the MIC of zeamines. Mutation of *desA* or *desB* led to only about an 8-fold decrease in MIC compared with that of the control strain (Table 1), while inactivation of *desC* could result in about a 32-fold decrease. A plausible explanation is that similar to its homolog *tolC* in E. coli (15), other proteins in strain EC1 are able to replace DesAB and form a functional transportation system with DesC to efflux zeamines. In addition, heterologous expression of *desABC* in E. coli resulted in only about a 2-fold increment in the MIC of zeamines, which was not comparable to the MIC changes when *desABC* were deleted in strain EC1. We first checked the possibility of whether the differences in GC content and codon usage of these two bacterial species affect the expression efficiency of *desABC* genes in E. coli. Our previous study showed that the GC content of the *D. zeae* EC1 genome is 53.43% (12), which is not identical but not substantially different from the GC content (50.8%) of E. coli strain K-12 (https://www.ncbi.nlm.nih.gov/genome/browse#!/prokaryotes/Escherichia%20coli%20K-12). Strain K-12 is the parental strain of E. coli strain DH5α used in this study. At the codon usage level, both bacteria have more or less similar codon usage patterns, except that the rarely used codon CUA in E. coli is a frequently used codon in the coding sequence of *desB* in *D. zeae* EC1 (Table S6). We then examined the potential toxic effect of overexpressed DesABC in E. coli, as other overexpressed membrane proteins commonly exhibit detrimental effects on bacterial growth (33). We found that E. coli growth was markedly retarded when DesABC were overexpressed (Fig. S4). Taken together, it is most likely that overexpressed DesABC membrane proteins affect the bacterial normal physiological functions and, hence, compromise the ability to withstand zeamines.

10.1128/mBio.00713-19.4 FIG S4 Heterologous expression of DesABC affects the growth of Escherichia coli. E. coli strain without plasmids (E. coli DH5α), E. coli strain with control plasmids [E. coli DH5α(pAmob, pBB)], and E. coli strain with *desABC* [E. coli DH5α(pAmob-*desC* pBB-*desAB*)] were grown to exponential phase in LB medium. Cell cultures were adjusted to an OD_600_ of about 0.5 and inoculated to LB medium at a ratio of 0.1%. Growth curves were determined at 37°C using Bioscreen-C (OY Growth Curves Ab Ltd., Helsinki, Finland) in a low-intensity model by monitoring the optical density of 600 nm. The experiments were individually performed twice. Data shown are the means from three replicates, and error bars indicate the standard deviations. Download FIG S4, PDF file, 0.3 MB. Copyright © 2019 Liang et al. 2019 Liang et al. This content is distributed under the terms of the Creative Commons Attribution 4.0 International license.

10.1128/mBio.00713-19.10 TABLE S6 Codon usage of the coding sequences of *desB* in Dickeya zeae EC1 and Escherichia coli O157:H7 strain EDL933. Download Table S6, DOCX file, 0.03 MB. Copyright © 2019 Liang et al. 2019 Liang et al. This content is distributed under the terms of the Creative Commons Attribution 4.0 International license.

Our data indicate that zeamine biosynthesis and DesABC-mediated resistance mechanisms are well coordinated by a sophisticated mechanism(s). The level of *desAB* gene expression was increased along with accumulation of zeamines (Fig. 3B and C and 4B), and deletion of *zmsA*, the gene essential for zeamine biosynthesis (4), caused an arrest of the transcriptional expression of *desAB* (Fig. 4B and C). Significantly, exogenous addition of zeamines to the *zmsA* mutant could boost *desAB* expression by more than 30-fold compared with that for water or solvent control (Fig. 4D). The findings thus demonstrated that in addition to their roles as phytotoxins and antibiotics (4, 5, 8–10), zeamines can also act as signals in modulation of gene expression (Fig. 4D). The signal role of zeamines in induction of *desAB* expression was further confirmed in *D. dadantii* strain 3937 (Fig. 4D), which does not contain a *zms* gene cluster. Considering the wild distribution of *zms* gene clusters and *desAB* homologs in *Dickeya* species and strains (Fig. 1 and Table S3) (8), we hypothesize that *Dickeya* species and strains have evolved a dedicated pathway to sense extracellular zeamines in self-protection against the detrimental effect of these antibiotics. The key regulators in this pathway might at least include a sensor or receptor protein that detects and responds to zeamines and a transcriptional regulator that modulates the expression of *desAB*. A gene that encodes a proposed two-component system sensor (NCBI accession number WP_029456608.1) was found near the *desAB* locus. However, inactivation of this gene did not affect the MIC of zeamines, which seems to preclude its potential link with the regulation of *desAB* (data not shown). In addition, given that the cellular levels of zeamines are important in induction of the zeamine resistance genes (Fig. 4D), several regulators known to be associated with the regulation of zeamine production and virulence, such as the acylhomoserine lactone (AHL) synthase ExpI (1), transcriptional regulator SlyA (3), and global regulator Fis (46), might also influence the transcriptional expression of *desAB* through modulating the production of zeamines or even more direct regulatory mechanisms, which demands further investigation.

In summary, this study documented a first resistance mechanism against zeamines, which are a new family of potent antibiotics with a broad spectrum of antimicrobial activities. This resistance mechanism is mediated by a novel and substrate-specific RND efflux pump, DesABC. Interestingly, this study also unveiled a signaling role of zeamines in modulation of *desAB* expression at the transcriptional level, which further expands our understanding about zeamines. In addition, the findings from this study suggest that *D. zeae* EC1 contains other mechanisms implicated in zeamine resistance besides the DesABC efflux system. This is evident as the MIC of zeamines for *D. zeae* EC1 was more than 500-fold higher than that for the zeamine-sensitive E. coli DH5α, whereas inactivation of *desC* in *D. zeae* EC1 led to only about a 32-fold decrease in the MIC of zeamines (Table 1). A thorough understanding of the zeamine resistance mechanisms and the cognate regulatory networks might pave the way for practical application of these potent antibiotics and also could provide new insight on the control and prevention of this important bacterial pathogen.

## MATERIALS AND METHODS

### Bacterial strains and growth conditions.

The strains and plasmids used in this study are listed in Table 2. *D. zeae* EC1 and derivatives were routinely grown at 28°C in Luria-Bertani (LB) medium, minimal medium (MM) [10.5 g K_2_HPO_4_, 4.5 g KH_2_PO_4_, 2.0 g (NH_4_)_2_SO_4_, 2.0 g mannitol, 2.0 g glycerol, 0.2 g MgSO_4_·7H_2_O, 0.01 g CaCl_2_, 0.005 g FeSO_4_·7H_2_O, and 0.002 g MnCl_2_·4H_2_O per liter, pH 7.0], or LS5 medium (5.25 g K_2_HPO_4_, 2.25 g KH_2_PO_4_, 10.0 g sucrose, 3.6 g NH_4_NO_3_, 1.0 g KCl, and 0.25 g MgSO_4_·7H_2_O per liter, pH 7.0) as indicated (29). E. coli strains were routinely grown at 37°C. The following antibiotics were supplemented when necessary: streptomycin, 50 μg/ml; kanamycin, 50 μg/ml; ampicillin, 50 μg/ml; chloramphenicol, 15 μg/ml.

**TABLE 2:** Bacterial strains and plasmids used in this study

Strain or plasmid	Relevant characteristic(s)^*a*^	Source or reference
Strains		
*Dickeya zeae*		
EC1	Wild-type strain of *D. zeae*	1
EC1(pDesAB_gfp_)	EC1 carry pDesAB_gfp_ vector, Kan^r^	This study
Δ*zmsA*	In-frame deletion of *zmsA* in EC1	Laboratory collection
Δ*zmsA*(pDesAB_gfp_)	Δ*zmsA* carrying pDesAB_gfp_ vector, Kan^r^	This study
Δ*zmsA*Δ*desA*	Δ*zmsA* carry the in-frame deletion of *desA*	This study
Δ*zmsA*Δ*desB*	Δ*zmsA* carrying the in-frame deletion of *desB*	This study
Δ*zmsA*Δ*desB*(pBB)	Δ*zmsA*Δ*desB* carrying pBBR1-MCS4 vector, Amp^r^	This study
Δ*zmsA*Δ*desB*(pBB-*desB*)	Δ*zmsA*Δ*desB* carrying pBB-*desB* vector, Amp^r^	This study
Δ*zmsA*Δ*desB*(pBB-*desB_3937_*)	Δ*zmsA*Δ*desB* carrying pBB-*desB_3937_* vector, Amp^r^	This study
Δ*zmsA*Δ*desC*	Δ*zmsA* carrying the in-frame deletion of *desC*	This study
Δ*zmsA*Δ*desC*(pBB-*desC*)	Δ*zmsA*Δ*desC* carrying pBB-*desC* vector, Amp^r^	This study
Δ*desB*	In-frame deletion of *desB* in EC1	This study
Δ*zmsR*	In-frame deletion of *zmsR* in EC1	Laboratory collection
Δ*zmsM*	In-frame deletion of *zmsM* in EC1	Laboratory collection
Escherichia coli		
DH5α	F^−^ φ80*lacZ*ΔM15 Δ(*lacZYA-argF*)*U169 endA1 recA1 hsdR17*(r_K_^−^ m_K_^+^) *supE44* λ^−^ *thi*-*1 gyrA96 relA1 phoA*	TransGen Biotech, China
DH5α(pBB, pAmob)	DH5α harboring both pBBR1-MCS4 (Amp^r^) and pAmob (Tet^r^, Chl^r^)	This study
DH5α(pBB-*desAB*, pAmob-*desC*)	DH5α harboring both pBB-*desAB* (Amp^r^) and pAmob-*desC* (Chl^r^)	This study
CC118	Host strain for replication of pKNG101 and derivate plasmids	Laboratory collection
HB101(pRK2013)	*thr leu thi recA hsdR hsdM pro*, Kan^r^	Laboratory collection
Dickeya dadantii		
3937(pDesAB_3937gfp_)	3937 carrying pDesAB_3937gfp_, Kan^r^	This study
Plasmids		
pKNG101	Suicide vector for gene in-frame deletion, Str^r^	Laboratory collection
pKNG-*desA*	pKNG101 harboring flanking region of *desA*	This study
pKNG-*desB*	pKNG101 harboring flanking region of *desB*	This study
pKNG-*desC*	pKNG101 harboring flanking region of *desC*	This study
pBB	Low-copy-number vector pBBR1-MCS4 with *lac* promoter, Amp^r^	Lab collection
pBB-*desB*	pBBR1-MCS4 harboring ORF of *desB* gene from EC1, Amp^r^	This study
pBB-*desB_3937_*	pBBR1-MCS4 harboring ORF of *desB* gene homolog from Dickeya dadantii 3937, Amp^r^	This study
pBB-*desAB*	pBBR1-MCS4 harboring ORF of *desAB* genes from EC1, Amp^r^	This study
pBB-*desC*	pBBR1-MCS4 harboring ORF of *desC* gene from EC1, Amp^r^	This study
pAmob	pACYC184 with *mob* region cloned from pBBR1-MCS4 inserting in BstZ17I restriction site, Tet^r^, Chl^r^	This study
pAmob-*desC*	pAmob harboring *desC* gene from EC1 under the control of tetracycline resistance gene promoter *tetO*, Chl^r^	This study
pPROBE-NT	Promoterless *gfp* transcriptional reporter plasmid, Kan^r^	43
pDesAB_gfp_	*gfp* transcriptional fusion with upstream region of *desAB* in EC1	This study
pDesAB_3937gfp_	*gfp* transcriptional fusion with upstream region of *desAB*_3937_ found in 3937	This study

a Abbreviations: Amp^r^, ampicillin resistance; Tet^r^, tetracycline resistance; Chl^r^, chloramphenicol resistance; Kan^r^, kanamycin resistance; Str^r^, streptomycin resistance.

### Construction of deletion and complementation strains.

Oligonucleotide primers used in this study are listed in Table S1 in the supplemental material. DNA manipulation was conducted by following methods described previously (5). Briefly, for gene in-frame deletion, fusion fragments containing the downstream and upstream regions of target genes were cloned into pKNG101 and transformed into E. coli CC118 for construction of gene in-frame deletion constructs. Triparental mating was performed by using wild-type strain EC1 or a *zmsA* in-frame deletion mutant as a recipient strain. Mutants were screened on an MM agar plate supplemented with 5% (wt/vol) sucrose, and desired deletions were confirmed by PCR and DNA sequencing. For complementation, the open reading frames (ORFs) of target genes were cloned into pBBR1-MCS4 and genes were expressed under the control of the *lac* promoter. The desired expression constructs were confirmed by PCR and DNA sequencing and introduced into corresponding mutants by triparental mating. The complementation strains were screened on MM agar plates containing ampicillin and verified by PCR. For construction of the strain expressing *desABC* heterologously, *desC* was cloned and expressed under the control of the *tetO* promoter in pAmob, while *desAB* were cloned into pBBR1-MCS4 and expressed under the control of the *lac* promoter. The resultant constructs, pBB-*desAB* and pAmob-*desC*, were cotransformed into E. coli DH5α for heterologous expression of the *desABC* efflux pump genes.

10.1128/mBio.00713-19.5 TABLE S1 Primers used in this study. Download Table S1, DOCX file, 0.03 MB. Copyright © 2019 Liang et al. 2019 Liang et al. This content is distributed under the terms of the Creative Commons Attribution 4.0 International license.

### Preparation of zeamines.

Overnight starter culture of wild-type strain EC1 grown in LB medium was inoculated (0.1%, vol/vol) into LS5 medium and grown at 28°C with rotation at 100 rpm for 48 h. The cells were then removed by centrifugation at 10,000 rpm at 4°C for 10 min. Approximately 10-liter supernatants were then passed slowly through the column containing 500 g of absorbent resin XAD7 (Sigma) at a flow rate of 1 ml/min by following the method described previously (4). The column was consecutively eluted with 2 liters of double-distilled H_2_O and 1 liter of methanol prior to elution with 2 liters of acetone to obtain the elutes containing zeamines. The acetone in the elutes was evaporated, and the residues were dissolved in methanol to obtain crude zeamine antibiotics. For confirmation, liquid chromatography-mass spectrometry (LC-MS) was performed using an Agilent 1260 infinity system connected to a Phenomenex Luna column (C_18_, 250 by 4.6 mm, 5 μm) coupled with a Bruker maxis Q-TOF mass spectrometer to identify three main zeamine antibiotics, zeamine, zeamine I, and zeamine II (Fig. S1). The crude zeamines were eluted with a gradient program of 5% to 95% (CH_3_CN supplemented with 1% formic acid in H_2_O) in 20 min at a flow rate of 1 ml/min. The mass spectrometer was employed in the positive ion mode, scanning from 100 to 2,000 *m/z*. The source conditions were set as the following: ESI source type, end plate offset at −500 V, capillary at 4,500 V, nebulizer gas (N_2_) at 0.8 bar, dry gas at 5.0 liters/min, and dry temperature at 180°C. The ion transfer condition was set as the following: collision cell RF of 800.0 Vpp. The antibiotic activity of zeamines was determined according to the method described below.

### Determination of MICs.

Determination of MICs of antibiotics in *D. zeae* and E. coli strains was conducted by following the protocol from the Clinical and Laboratory Standards Institute (35). Briefly, 2-fold dilutions of antibiotics in LB were added to 96-well plates, and fresh bacterial culture in LB medium was added to obtain about 2.0 × 10^5^ CFU/ml in each well. The plates were incubated at 28°C or 37°C for 18 h, and the minimum antibiotic concentration with no visible cell growth was defined as the MIC.

### Construction of phylogenic tree.

A total of 59 amino acid sequences obtained from NCBI (Table S3) were used in construction of the phylogenic tree, including 51 sequences found by blastp search, with the highest total score from *Dickeya* species and other proteobacterial species, 3 sequences from *S. plymuthica* strains containing homologs of the *zms* gene cluster, and 5 sequences with known substrate profiles. The protein sequences were aligned using MAFFT v7.402 (36) in the “einsi” mode, and the multiple-sequence alignment (MSA) was filtered for columns with high proportions of missing data using trimAl v1.4 (37) with the “-gappyout” option. The filtered MSA was analyzed by IQ-TREE v1.6.5 (38) to first perform a model selection with the “-MF” option (39), and then we carried out a maximum-likelihood tree inference under the best-fit model (“LG+R5”) with 1,000 ultrafast bootstrap support (40).

### Survival assay.

The survival assay was conducted by following the kill curve method described previously, with minor modifications (41). Briefly, fresh bacterial cultures in LB medium at exponential phase were collected and adjusted to an OD_600_ of about 1.0 (±0.05). Cells from 1 ml culture were harvested (4,000 rpm, 4°C, 5 min) and washed twice with LS5 salts (LS5 medium without sucrose). Bacterial cells were then resuspended in LS5 salts and added to 96-well plates with LS5 salts containing zeamines. The final concentration of zeamines in the assay was at 2-fold the MIC of the *zmsA*-*desB* mutant. The plates were incubated at 28°C with agitation at 200 rpm, and the survivors were determined at specific time points by plating appropriate bacterial dilutions on LB plates.

### Growth kinetics assay measured in the flasks with LS5 medium.

Bacterial growth curves in LS5 medium were measured by following the procedures described for zeamine preparation, with minor modifications. Briefly, overnight cell cultures were adjusted to an OD_600_ of about 0.5 (±0.05) before inoculation at a ratio of 0.1%, vol/vol, and flasks were kept at 28°C with shaking at 150 rpm. The optical density at 600 nm was measured at different time points, as indicated, by the NanoDrop 2000c (Thermo Fisher Scientific, MA, USA) with proper dilutions when necessary.

### Zeamine production assay.

The assay of zeamine production was conducted by following a method described previously, with minor modifications (4). Briefly, 25 ml LB agar was poured in 10- by 10-cm square plates and overlaid with 7.5 ml 1% (wt/vol) agarose containing about 1.5 × 10^8^ fresh E. coli DH5α cells. The wells, at 4-mm diameter, were punched in the plate, and 30 μl of cell-free supernatants (filter sterilized with a 0.22-μm pore filter) were added in each well. The plates were incubated at 37°C for 24 h, and the inhibition zone around the wells was measured. For semiquantification, one unit of zeamines was defined as the amount that could generate a 2-mm-diameter inhibitory zone around the well. The number of zeamine units per milliliter was calculated by multiplying the units of zeamines calculated from the bioassay by the fold change of sample volume used in the test (30 μl) to the total volume (1 ml).

### Construction of transcriptional fusion construct pDesAB_gfp_ and pDesAB_3937gfp_ and flow cytometry analysis.

As there is only a 20-bp interval region located between the ORFs of *desA* and *desB*, the *desAB* genes were considered to be located in the same operon. The promoter region of *desAB* genes was predicted by using the online tool provided by BPROM (42) (http://www.softberry.com/berry.phtml?topic=bprom&group=programs&subgroup=gfindb). The 204-bp DNA fragment upstream of the ORF of *desAB* was amplified using the primer pair P-desAB-F and P-desAB-R (Table S1) and ligated into the promoterless *gfp*-reporter plasmid pPROBE-NT (43) for generation of the construct pDesAB_gfp._ pDesAB_gfp_ and pPROBE-NT were separately mobilized into wild-type EC1 and the *zmsA* mutant by triparental mating with the helper strain HB101(pRK2013) to construct EC1(pDesAB_gfp_) and Δ*zmsA*(pDesAB_gfp_). 3937(pDesAB_3937gfp_) was constructed by a similar method. Expression of *desAB* and *desAB_3937_* was analyzed by monitoring the average fluorescence of 50,000 cells when bacteria were grown in flasks with LS5 medium at different time points by a CytoFLEX flow cytometer (Beckman Coulter, Brea, CA, USA) by following the method previously described (44).

### RNA extraction and real-time qPCR analysis.

Bacterial cells were cultured and harvested at an OD_600_ of about 1.5 (±0.05). RNA extraction was performed using the RiboPure RNA purification kit, bacteria (Thermo Fisher Scientific, MA, USA), by following the manufacturer’s instructions. The purity of RNA was determined by gel electrophoresis, and the *A*_260_/*A*_280_ and *A*_260_/*A*_230_ ratios were determined using a NanoDrop 2000c (Thermo Fisher Scientific, MA, USA). In qPCR analysis, an aliquot of 300 ng RNA sample was used for genomic DNA elimination and cDNA synthesis by a FastKing RT kit (with gDNase) (Tiangen Biotech, Co., Ltd., Beijing, China) by following the manufacturer’s protocol. Specific primers for the *desC*, *desB*, and 16S rRNA genes (Table S1) were designed by AlleleID 6.0 (PRIMER Biosoft). The housekeeping gene 16S rRNA was used as a reference. The PCR efficiency of each gene was determined using five DNA standards at different concentrations (10, 1, 0.1, 0.01, and 0.001 μg/ml). The qPCR analysis was conducted on a Quantstudio 6 Flex system using PowerUp SYBR green master mix (Thermo Fisher Scientific) with the following cycle profile: 1 cycle at 50°C for 2 min and 95°C for 2 min, followed by 40 cycles at 95°C for 15 s, 57°C for 15 s, and 72°C for 30 s. The experiment was repeated three times, each time with triplicates. Data were analyzed using the 2^−ΔΔCT^ method as previously described (45).

### Statistical analysis.

Experiments were individually performed at least twice with three replicates each time. Data shown are the means from three replicates, and error bars indicated the standard deviations or standard errors. Statistical comparison was performed by using Student's *t* test in GraphPad Prism 5.0 software (GraphPad, La Jolla, CA). A *P* value of less than 0.05 was considered significant.

### Data availability.

The genome sequence of *D. zeae* EC1 is accessible in NCBI under accession number NZ_CP006929.1. The amino acid sequence of TolC in E. coli K-12 AG100 is accessible in NCBI under accession number WP_000735278.1.
