# Supplementary material for: A Substrate-Activated Efflux Pump, DesABC, Confers Zeamine Resistance to Dickeya zeae
Source: mBio. 2019 May 28;10(3):e00713-19. doi: 10.1128/mBio.00713-19 (PMC6538784; doi:10.1128/mBio.00713-19)
Supplement: FIG S1 [file mBio.00713-19-sf001.pdf]

A

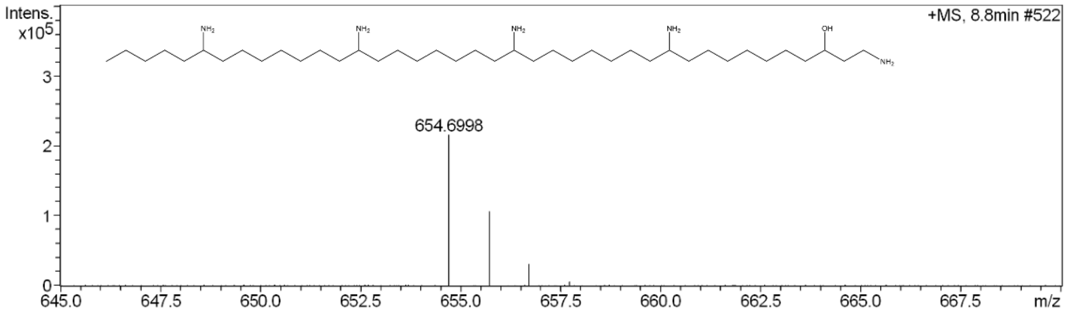


| Meas. m/z | # | Formula | Score | m/z | err [ppm] | err [mDa] | mSigma | rdb | e⁻ Conf | N-Rule |
|---|---|---|---|---|---|---|---|---|---|---|
| 654.6998 | 1 | C 40 H 88 N 5 O | 100.00 | 654.6983 | -2.2 | -1.4 | 24.0 | -0.5 | even | ok |

B

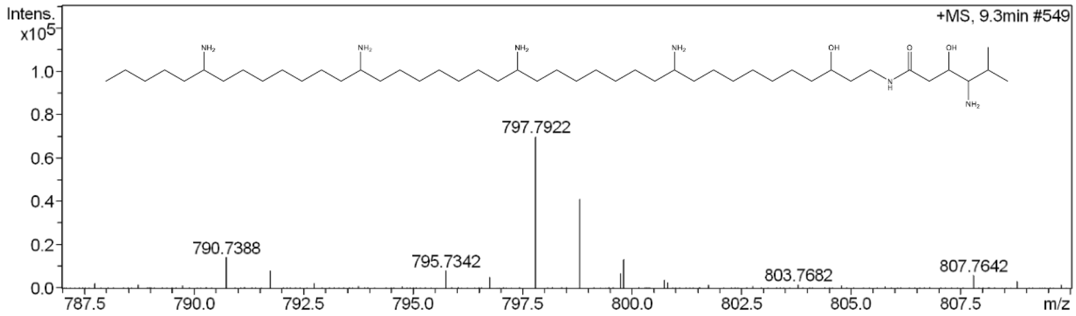


| Meas. m/z | # | Formula | Score | m/z | err [ppm] | err [mDa] | mSigma | rdb | e⁻ Conf | N-Rule |
|---|---|---|---|---|---|---|---|---|---|---|
| 797.7922 | 1 | C 47 H 101 N 6 O 3 | 100.00 | 797.7930 | 1.0 | 0.8 | 29.2 | 0.5 | even | ok |

C

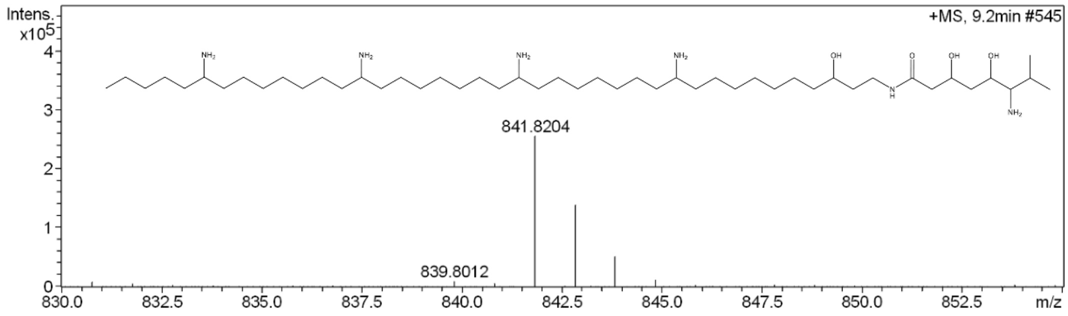


| Meas. m/z | # | Formula | Score | m/z | err [ppm] | err [mDa] | mSigma | rdb | e⁻ Conf | N-Rule |
|---|---|---|---|---|---|---|---|---|---|---|
| 841.8204 | 1 | C 49 H 105 N 6 O 4 | 65.06 | 841.8192 | -1.5 | -1.2 | 21.8 | 0.5 | even | ok |

**FIG S1** High resolution mass spectrum of zeamine II (A) zeamine I (B) and zeamine (C) detected by LC-MS in purified zeamines. The structure was drawn by using the software ChemBioDraw Ultra 14.0 (Cambridgesoft).
