# Supplementary material for: A Substrate-Activated Efflux Pump, DesABC, Confers Zeamine Resistance to Dickeya zeae
Source: mBio. 2019 May 28;10(3):e00713-19. doi: 10.1128/mBio.00713-19 (PMC6538784; doi:10.1128/mBio.00713-19)
Supplement: TABLE S2 [file mBio.00713-19-st002.docx]

**TABLE S2** Identity and similarity of the transporter genes within and adjacent to the *zms* gene clusters in *Dickeya* species and *Serratia plymuthica* strains

| Strain  (Sequence accession number in NCBI) | Identity (similarity) in amino acid level to the corresponding  homolog in strain EC1 | | | | | | | |
| --- | --- | --- | --- | --- | --- | --- | --- | --- |
|  | *zmsP* | *zmsQ* | *zmR* | *zmsL* | *zmsM* | *zmN* | *desB* | *desA* |
| *Dickeya zeae* DZQ  (NZ_APMV01000018.1) | 99  (99) | 100  (100) | 100  (100) | 100  (100) | 100  (100) | 99  (99) | 99  (100) | 99  (100) |
| *Dickeya* sp. MK7  (NZ_CM001984.1) | 89  (92) | 94  (95) | 91  (95) | 92  (96) | 87  (93) | 81  (88) | 89  (94) | 83  (90) |
| *Dickeya solani* IPO2222  (NZ_CM001859.1) | 88  (93) | 94  (96) | 91  (96) | 92  (96) | 86  (91) | 80  (87) | 89  (93) | 82  (89) |
| *Serratia plymuthica* AS9  (NC_015567.1) | 64  (79) | 69  (79) | 71  (84) | 73  (85) | 69  (82) | 56  (71) | - | - |
| *Serratia plymuthica* RHV1  (NZ_ARWD01000001.1) | 64  (79) | 69  (78) | 71  (84) | 73  (86) | 69  (82) | 57  (72) | - | - |
| *Serratia plymuthica* S13  (NC_021659.1) | 64  (79) | 69  (78) | 71  (84) | 73  (86) | 69  (82) | 57  (72) | - | - |

Symbol: “-” means that the homolog is not present in the corresponding region of the genome.
