# Supplementary material for: A Substrate-Activated Efflux Pump, DesABC, Confers Zeamine Resistance to Dickeya zeae
Source: mBio. 2019 May 28;10(3):e00713-19. doi: 10.1128/mBio.00713-19 (PMC6538784; doi:10.1128/mBio.00713-19)
Supplement: FIG S2 [file mBio.00713-19-sf002.pdf]

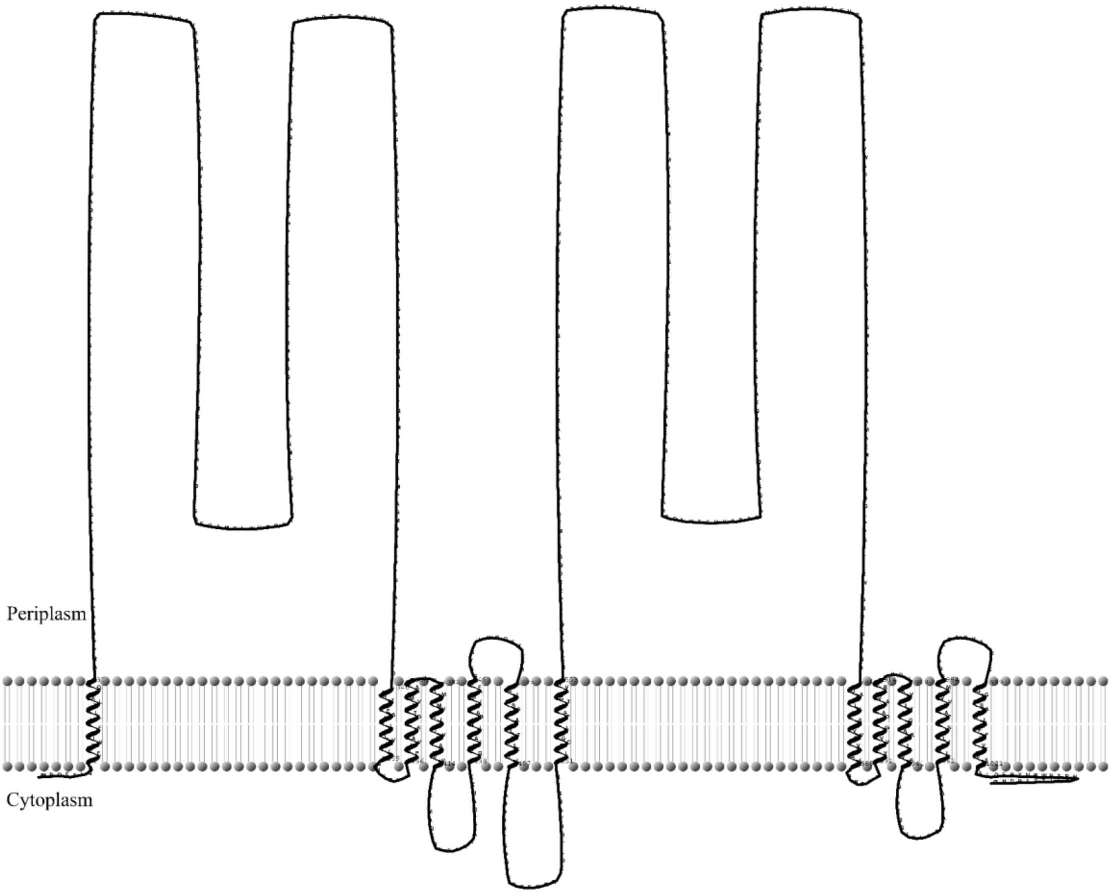

FIG S2 Predicted topological structure of DesB in cell membrane. The TMHMM (http://www.cbs.dtu.dk/services/TMHMM/) was used to analyze DesB amino acid and the result was output in TMRPRES2D software.
