# Supplementary material for: A Substrate-Activated Efflux Pump, DesABC, Confers Zeamine Resistance to Dickeya zeae
Source: mBio. 2019 May 28;10(3):e00713-19. doi: 10.1128/mBio.00713-19 (PMC6538784; doi:10.1128/mBio.00713-19)
Supplement: FIG S3 [file mBio.00713-19-sf003.docx]

**
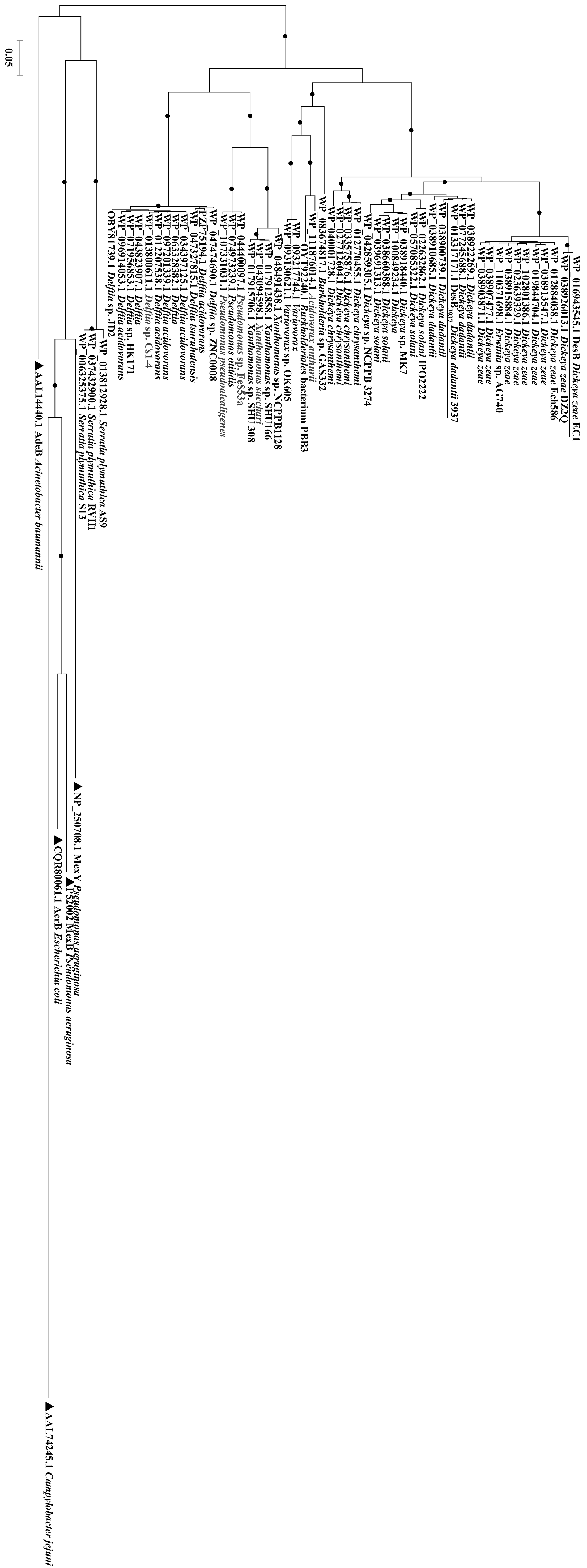
**

**FIG S3** Phylogenic relationship of DesB with the inner membrane proteins of other RND efflux pumps. The black dots show the bootstrap support higher than 95%. The underlines show the positions of DesB from *Dickeya zeae* EC1 and DesB_3937_ from *Dickeya dadantii* 3937. The black triangles show the proteins whose substrate profiles were determined previously.
