# Supplementary material for: A Substrate-Activated Efflux Pump, DesABC, Confers Zeamine Resistance to Dickeya zeae
Source: mBio. 2019 May 28;10(3):e00713-19. doi: 10.1128/mBio.00713-19 (PMC6538784; doi:10.1128/mBio.00713-19)
Supplement: TABLE S3 [file mBio.00713-19-st003.docx]

**TABLE S3** Characteristics of RND family genes used in phylogenic study

| Number | Protein description^a^ | | Accession number in NCBI | Identity (similarity) with *desB* in amino acid level (%) |
| --- | --- | --- | --- | --- |
| **DesB homologs found in NCBI** | | | | |
| 1 | | DesB  [*Dickeya zeae* EC1] | WP_016943545.1 | 100 (100) |
| 2 | | multidrug efflux RND transporter permease subunit  [*Dickeya zeae* DZ2Q] | WP_038926013.1 | 99 (100) |
| 3 | | multidrug efflux RND transporter permease subunit  [*Dickeya zeae* Ech586] | WP_012884038.1 | 97 (98) |
| 4 | | multidrug efflux RND transporter permease subunit  [*Dickeya zeae*] | WP_038913547.1 | 97 (98) |
| 5 | | multidrug efflux RND transporter permease subunit  [*Dickeya zeae*] | WP_038907477.1 | 97 (98) |
| 6 | | multidrug efflux RND transporter permease subunit  [*Dickeya zeae*] | WP_102801386.1 | 97 (98) |
| 7 | | multidrug efflux RND transporter permease subunit  [*Erwinia* sp. AG740] | WP_110371698.1 | 97 (98) |
| 8 | | multidrug efflux RND transporter permease subunit  [*Dickeya zeae*] | WP_038903871.1 | 97 (98) |
| 9 | | multidrug efflux RND transporter permease subunit  [*Dickeya zeae*] | WP_023639329.1 | 97 (98) |
| 10 | | multidrug efflux RND transporter permease subunit  [*Dickeya zeae*] | WP_019844704.1 | 96 (97) |
| 11 | | multidrug efflux RND transporter permease subunit  [*Dickeya zeae*] | WP_038915884.1 | 96 (97) |
| 12 | | multidrug efflux RND transporter permease subunit  [*Dickeya dadantii*] | WP_038922269.1 | 89 (94) |
| 13 | | multidrug efflux RND transporter permease subunit  [*Dickeya dadantii*] | WP_038900739.1 | 89 (94) |
| 14 | | multidrug efflux RND transporter permease subunit  [*Dickeya* sp. MK7] | WP_038918440.1 | 89 (94) |
| 15 | | DesB_3937_  [*Dickeya dadantii* 3937] | WP_013317171.1 | 89 (94) |
| 16 | | multidrug efflux RND transporter permease subunit  [*Dickeya solani*] | WP_038660388.1 | 89 (94) |
| 17 | | multidrug efflux RND transporter permease subunit  [*Dickeya solani*] | WP_039691313.1 | 89 (94) |
| 18 | | multidrug efflux RND transporter permease subunit  [*Dickeya dadantii*] | WP_077245688.1 | 89 (94) |
| 19 | | multidrug efflux RND transporter permease subunit  [*Dickeya* sp. NCPPB 3274] | WP_042859305.1 | 89 (94) |
| 20 | | multidrug efflux RND transporter permease subunit  [*Dickeya*] | WP_100849234.1 | 89 (94) |
| 21 | | multidrug efflux RND transporter permease subunit  [*Dickeya solani* IPO2222] | WP_022632862.1 | 89 (93) |
| 22 | | multidrug efflux RND transporter permease subunit  [*Dickeya solani*] | WP_057085322.1 | 89 (93) |
| 23 | | multidrug efflux RND transporter permease subunit  [*Dickeya chrysanthemi*] | WP_012770455.1 | 88 (93) |
| 24 | | multidrug efflux RND transporter permease subunit  [*Dickeya chrysanthemi*] | WP_040001728.1 | 89 (93) |
| 25 | | multidrug efflux RND transporter permease subunit  [*Dickeya chrysanthemi*] | WP_027712604.1 | 88 (93) |
| 26 | | multidrug efflux RND transporter permease subunit  [*Dickeya chrysanthemi*] | WP_033575876.1 | 88 (93) |
| 27 | | multidrug efflux RND transporter permease subunit  [*Dickeya dadantii*] | WP_038910685.1 | 89 (93) |
| 28 | | multidrug efflux RND transporter permease subunit [*Burkholderia* sp. GAS332] | WP_083674817.1 | 78 (88) |
| 29 | | multidrug efflux RND transporter permease subunit  [*Acidovorax anthurii*] | WP_111876014.1 | 76 (86) |
| 30 | | multidrug efflux RND transporter permease subunit  [*Xanthomonas* sp. NCPPB1128] | WP_048491438.1 | 76 (86) |
| 31 | | multidrug efflux RND transporter permease subunit  [*Variovorax*] | WP_093217744.1 | 76 (87) |
| 32 | | multidrug efflux RND transporter permease subunit  [*Pseudomonas* sp. FeS53a] | WP_044400097.1 | 75 (85) |
| 33 | | multidrug efflux RND transporter permease subunit [*Variovorax* sp. OK605] | WP_093130621.1 | 76 (87) |
| 34 | | multidrug efflux RND transporter permease subunit [*Pseudomonas otitidis*] | WP_074973239.1 | 75 (85) |
| 35 | | multidrug efflux RND transporter permease subunit  [*Delftia* sp. ZNC0008] | WP_047474690.1 | 77 (87) |
| 36 | | multidrug efflux RND transporter permease subunit  [*Delftia acidovorans*] | WP_034397125.1 | 77 (87) |
| 37 | | multidrug efflux RND transporter permease subunit [*Xanthomonas* sp. SHU166] | WP_017912858.1 | 76 (86) |
| 38 | | acriflavine resistance protein B  [*Delftia* sp. JD2] | OBY81739.1 | 77 (87) |
| 39 | | multidrug efflux RND transporter permease subunit  [*Delftia*] | WP_043823907.1 | 77 (87) |
| 40 | | multidrug efflux RND transporter permease subunit  [*Delftia*] | WP_063328382.1 | 77 (87) |
| 41 | | multidrug efflux RND transporter permease subunit  [*Delftia acidovorans*] | WP_012207538.1 | 77 (87) |
| 42 | | multidrug efflux RND transporter permease subunit  [*Delftia* sp. HK171] | WP_071956853.1 | 77 (87) |
| 43 | | multidrug efflux RND transporter permease subunit  [*Delftia acidovorans*] | WP_097201339.1 | 77 (87) |
| 44 | | multidrug efflux RND transporter permease subunit  [*Xanthomonas sacchari*] | WP_043094598.1 | 76 (86) |
| 45 | | multidrug efflux RND transporter permease subunit  [*Burkholderiales* bacterium PBB3] | OYT92240.1 | 75 (86) |
| 46 | | multidrug efflux RND transporter permease subunit  [*Xanthomonas* sp. SHU 308] | WP_017915706.1 | 76 (86) |
| 47 | | multidrug efflux RND transporter permease subunit  [*Delftia acidovorans*] | PZP75194.1 | 77 (87) |
| 48 | | multidrug efflux RND transporter permease subunit  [*Delftia acidovorans*] | WP_096914053.1 | 77 (87) |
| 49 | | multidrug efflux RND transporter permease subunit  [*Pseudomonas pseudoalcaligenes*] | WP_107331031.1 | 75 (85) |
| 50 | | multidrug efflux RND transporter permease subunit  [*Delftia* sp. Cs1-4] | WP_013800611.1 | 77 (87) |
| 51 | | multidrug efflux RND transporter permease subunit  [*Delftia tsuruhatensis*] | WP_047327815.1 | 77 (87) |
| **RND family proteins found in** ***Serratia*** ***plymuthica* strains with *zms* gene clusters** | | | | |
| 52 | | multidrug efflux RND transporter permease subunit  [*Serratia plymuthica* AS9] | WP_013812928.1 | 65 (80) |
| 53 | | multidrug efflux RND transporter permease subunit  [*Serratia plymuthica* RVH1] | WP_037432900.1 | 65 (80) |
| 54 | | multidrug efflux RND transporter permease subunit  [*Serratia plymuthica* S13] | WP_006325375.1 | 65 (80) |
| **RND family proteins with substrate profile being determined** | | | | |
| 55 | | AdeB  [*Acinetobacter baumannii*] | AAL14440.1 | 63 (80) |
| 56 | | MexY  [*Pseudomonas aeruginosa*] | NP_250708.1 | 51 (67) |
| 57 | | MexB  [*Pseudomonas aeruginosa*] | P52002 | 50 (68) |
| 58 | | AcrB  [*Escherichia coli*] | CQR80061.1 | 50 (69) |
| 59 | | CmeB  [*Campylobacter jejuni*] | AAL74245.1 | 38 (61) |

^a^ Data is derived from NCBI using blastp. Fifty-one DesB homologous proteins in NCBI with the highest total score were chosen except one sequence (accession number in NCBI is WP_047643084.1) without the dominant feature of RND family inner membrane protein (1). Three DesB homologous proteins with the highest total score found by blastp in the genome of *S. plymuthica* strains with *zms* gene clusters, and five inner membrane proteins of RND efflux systems with known substrate profiles were also chosen for comparative analysis.

**REFERENCES:**

1. Eicher T, Cha HJ, Seeger MA, Brandstätter L, El-Delik J, Bohnert JA, Kern WV, Verrey F, Grütter MG, Diederichs K, Pos KM. 2012. Transport of drugs by the multidrug transporter AcrB involves an access and a deep binding pocket that are separated by a switch-loop. Proc Natl Acad Sci U S A 109:5687-5692
