# Supplementary material for: A Substrate-Activated Efflux Pump, DesABC, Confers Zeamine Resistance to Dickeya zeae
Source: mBio. 2019 May 28;10(3):e00713-19. doi: 10.1128/mBio.00713-19 (PMC6538784; doi:10.1128/mBio.00713-19)
Supplement: TABLE S4 [file mBio.00713-19-st004.docx]

**TABLE S4** Classes and targets of antibiotics

| Antibiotics | Classes^a^ | Target^b^ |
| --- | --- | --- |
| ampicillin | β-lactams | cell wall synthesis |
| tetracycline | tetracycline | protein synthesis |
| kanamycin | aminoglycosides | protein synthesis |
| gentamicin | aminoglycosides | protein synthesis |
| streptomycin | aminoglycosides | protein synthesis |
| chloramphenicol | phenicols | protein synthesis |
| polymyxin B | lipopetides | cell membrane |

^a,b^ Reference: (1, 2)

**REFERENCES**

1. Clinical and Laboratory Standards Institute. 2012. Methods for dilution antimicrobial susceptibility tests for bacteria that grow aerobically. CLSI document M07-A9. Clinical and Laboratory Standards Institute, Wayne, PA
2. Walsh C. 2003. Where will new antibiotics come from? Nat Rev Microbiol 1:65-70.
