# Supplementary material for: A Substrate-Activated Efflux Pump, DesABC, Confers Zeamine Resistance to Dickeya zeae
Source: mBio. 2019 May 28;10(3):e00713-19. doi: 10.1128/mBio.00713-19 (PMC6538784; doi:10.1128/mBio.00713-19)
Supplement: TABLE S5 [file mBio.00713-19-st005.docx]

**TABLE S5** Antibiotic susceptibility of the *desB* mutant, *desAB* overexpression strain and their parental strains

|  | MIC(μg/ml) ^a^ | | | | | | |
| --- | --- | --- | --- | --- | --- | --- | --- |
|  | AMP | TET | KAN | GEN | STR | CHL |  |
| ∆*zmsA* | ＜0.20 | 0.13 | 12.5 | 20 | 20 | 0.63 |  |
| ∆*zmsA*∆*desB* | ＜0.20 | 0.13 | 12.5 | 20 | 20 | 0.63 |  |
| EC1 | ND | 0.13 | 12.5 | 20 | 20 | 0.63 |  |
| EC1 (pBB) | ND | 0.13 | 12.5 | 20 | 20 | 0.63 |  |
| EC1 (pBB-desAB) | ND | 0.13 | 12.5 | 20 | 20 | 0.63 |  |

^a^Abbreviations: AMP: ampicillin; TET: tetracycline; KAN: kanamycin; GEN: gentamicin; STR: streptomycin; CHL: chloramphenicol; ND: no determined.
