# Supplementary material for: A Substrate-Activated Efflux Pump, DesABC, Confers Zeamine Resistance to Dickeya zeae
Source: mBio. 2019 May 28;10(3):e00713-19. doi: 10.1128/mBio.00713-19 (PMC6538784; doi:10.1128/mBio.00713-19)
Supplement: FIG S4 [file mBio.00713-19-sf004.pdf]

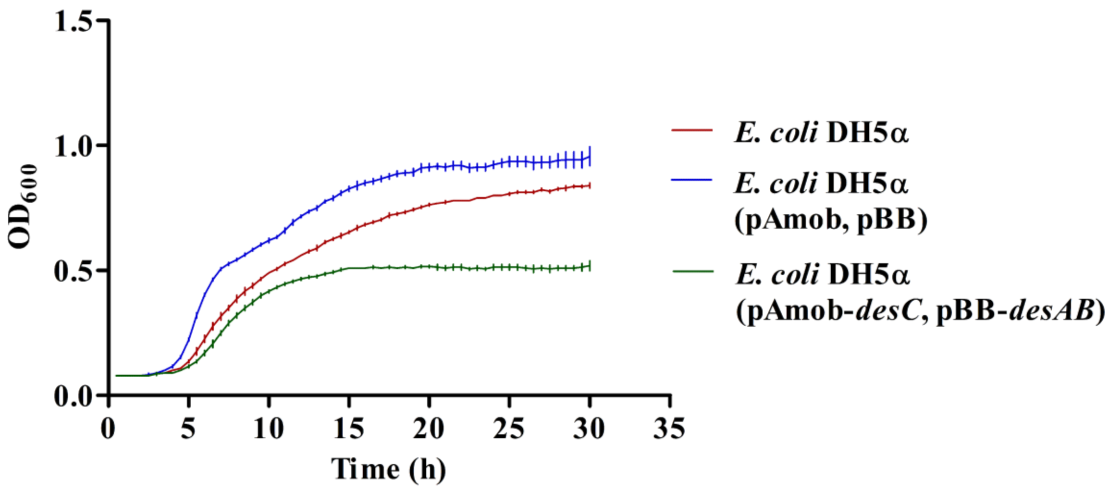

FIG S4 Heterologous expression of DesABC affects the growth of *Escherichia coli*. *E. coli* strain without plasmids (*E. coli* DH5α), *E. coli* strain with control plasmids (*E. coli* DH5α (pAmob, pBB) and *E. coli* strain with DesABC (*E. coli* DH5α (pAmob-*desC* pBB-*desAB*)) were grown to the exponential phase in LB medium. Cell cultures were adjusted to optical density about 0.5 (±0.05) and inoculated to LB medium at a ratio of 0.1%. Growth curves were determined at 37°C using Bioscreen-C (OY Growth Curves Ab Ltd, Helsinki, Finland) in a low-intensity model by monitoring the optical density of 600 nm. The experiments were individually performed twice. Data shown were the means of three replicates and error bars indicate the standard deviations.
