# Supplementary material for: A Substrate-Activated Efflux Pump, DesABC, Confers Zeamine Resistance to Dickeya zeae
Source: mBio. 2019 May 28;10(3):e00713-19. doi: 10.1128/mBio.00713-19 (PMC6538784; doi:10.1128/mBio.00713-19)
Supplement: TABLE S6 [file mBio.00713-19-st006.docx]

**TABLE S6** Codon usage of the coding sequences of *desB* in *Dickeya zeae* EC1 and *Escherichia coli* O157:H7 strain EDL933

| Rank^a^ | Codon usage of the coding sequences | |
| --- | --- | --- |
|  | *DesB* in *D. zeae* EC1 | *E. coli* O157:H7 strain EDL933 |
| 1 | CUG | CUG |
| 2 | GUG | GAA |
| 3 | GCC | AAA |
| 4 | ACC | GAU |
| 5 | GCG | GCG |
| 6 | AUG | AUU |
| 7 | AUC | CAG |
| 8 | GAA | GGC |
| 9 | GGC | AUG |
| 10 | CAG | GUG |
| 11 | UUU | GCC |
| 12 | CAA | GGU |
| 13 | CCG | AUC |
| 14 | AAC | ACC |
| 15 | GGU | CCG |
| 16 | UUG | UUU |
| 17 | AUU | AAC |
| 18 | CGU | CGC |
| 19 | GAU | GCA |
| 20 | UUC | CGU |
| 21 | UCG | GAC |
| 22 | CGC | AAU |
| 23 | GUA | GAG |
| 24 | GCA | GUU |
| 25 | GUC | UAU |
| 26 | **CUA** | AGC |
| 27 | ACG | UUC |
| 28 | AGC | GCU |
| 29 | CUC | UGG |
| 30 | GAC | ACG |
| 31 | GAG | GUC |
| 32 | AAA | CAA |
| 33 | GCU | UUA |
| 34 | ACA | UUG |
| 35 | UCC | CAU |
| 36 | GGG | UAC |
| 37 | CCA | GGG |
| 38 | UAC | CUU |
| 39 | CCC | AAG |
| 40 | UAU | GUA |
| 41 | UCU | CUC |
| 42 | CGG | CAC |
| 43 | GUU | AGU |
| 44 | UCA | ACU |
| 45 | UUA | UCC |
| 46 | CCU | GGA |
| 47 | GGA | UCG |
| 48 | UGG | UCU |
| 49 | AUA | CCA |
| 50 | AAG | UCA |
| 51 | AAU | ACA |
| 52 | AGU | CCU |
| 53 | CAC | UGC |
| 54 | CAU | CGG |
| 55 | AGA | CCC |
| 56 | CGA | AUA |
| 57 | CUU | UGU |
| 58 | ACU | **CUA** |
| 59 | AGG | CGA |
| 60 | UGC | AGA |
| 61 | UGU | AGG |

^a^: The rank of each codons was arranged by their frequency in the total 1046 codons in *desB* coding sequence or their frequency in coding sequences of *E. coli* O157:H7 strain EDL933 found in Codon Usage Database (1) (http://www.kazusa.or.jp/codon/cgi-bin/showcodon.cgi?species=155864). The red bold showed the position of the codon CUA in the coding sequences of *desB* in *D. zeae* EC1 and *E. coli* O157:H7 strain EDL933. Condon usage of *E. coli* O157:H7 strain EDL933 was employed for representing the codon usage of *E. coli* derivatives.

**REFERENCES**

1. Nakamura Y, Gojobori T, Ikemura T. 2000. Codon usage tabulated from international DNA sequence databases: status for the year 2000. Nucleic Acids Res 28:292.
