# Supplementary material for: A Substrate-Activated Efflux Pump, DesABC, Confers Zeamine Resistance to Dickeya zeae
Source: mBio. 2019 May 28;10(3):e00713-19. doi: 10.1128/mBio.00713-19 (PMC6538784; doi:10.1128/mBio.00713-19)
Supplement: TABLE S1 [file mBio.00713-19-st001.docx]

**TABLE S1** Primers used in this study

| Name | Sequence | | Description | |
| --- | --- | --- | --- | --- |
| **Primers for generation of fusion fragment for in-frame gene deletion** | | | | |
| desA-1 | 5’-cgggatccAACGGGCAATCGGCAACTGG-3’ | | | *desA* in-frame deletion |
| desA-2 | 5’-CAGCGGTAAGAACGGCGGAAGTGCTGCTGCGGATTCTGGT-3’ | | |  |
| desA-3 | 5’-ACCAGAATCCGCAGCAGCACTTCCGCCGTTCTTACCGCTG-3’ | | |  |
| desA-4 | 5’-cgggatccCGGAAACCGCACACAGAAAA-3’ | | |  |
| desB-1 | 5’-ggactagtCCAATGAAGAGGGGAGCGAT-3’ | | | *desB* in-frame deletion |
| desB-2 | 5’-CGGAACAGGCGATGCGTATCGTGATGTTGGTGGTGCCGTT-3’ | | |  |
| desB-3 | 5’-AACGGCACCACCAACATCACGATACGCATCGCCTGTTCCG-3’ | | |  |
| desB-4 | 5’-ggactagtCCAATACCGCATCCGCACTG-3’ | | |  |
| desC-1 | 5’-ggactagtGGACGGATTAGTGCGAGAGC-3’ | | | *desC* in-frame deletion |
| desC-2 | 5’-ACCAACAGGCCAAAACGACCACCACCACCAAAGCCAGACC-3’ | | |  |
| desC-3 | 5’-GGTCTGGCTTTGGTGGTGGTGGTCGTTTTGGCCTGTTGGT-3’ | | |  |
| desC-4 | 5’-ggactagtATAATCGGGTGCGGGAAAGT-3’ | | |  |
| **Primers for complementation and heterologous expression** | | | | |
| C-desB-F | 5’-aaaactgcaggagctcATCGCTCCCCAATGAAGAGG-3’ | | | *desB* complementation or *desAB* heterologous expression |
| C-desB-R | 5’-cccaagcttGCCAATACCGCATCCGCACT-3’ | | |  |
| C-desA-R | 5’-cccaagcttCAGCGATAAGCACCAATCCA-3’ | | |  |
| C-desC-F  (pBB) | 5’-aaaactgcaggagctcAGGAGAAGGGGGAATGGCTT-3’ | | | *desC* complementation or heterologous expression |
| C-desC-F (pAmob) | 5’-aaaactgcaggtcgacGGAGAAGGGGGAATGGCTTG-3’ | | |  |
| C-desC-R | 5’-cgcggatccCGGATAATCGGGTGCGGGAA-3’ | | |  |
| **Primers for cloning *mob* region from pBBR1-MCS4 to pACYC184** | | | | |
| mob-b-F | 5’-aaaactgcaggtatacCGGGGAGTCAGGCAACTATG-3’ | | |  |
| mob-b-R | 5’-CGTAGGCGGTCACGACTTTG-3’ | | |  |
| **Primers for amplification of the *desAB* and *desAB*_3937_ upstream region for construction of pDesAB_gfp_ and pDesAB_3937gfp_** | | | | |
| P-desAB-F | 5’-cgggatccCCGACAGGCCGGTAACATGC-3’ | pDesAB_gfp_ construction | | |
| P-desAB-R | 5’-cccaagcttAGCAATGGGCATGGATTGCG-3’ |  |  |  |
| P-desAB_3937_-F | 5’-gtacccggggatccCTTGGTGTTCATATCACGTGG-3’ | pDesAB_3937gfp_ construction | | |
| P- desAB_3937_-R | 5’-TTGGGGATCGGaagcttAGCGCGGTCAGCATAATCAC-3’ |  |  |  |
| **Primers for qPCR analysis** | | | | |
| qPCR-16S-F | 5’-CCAGGTGTAGCGGTGAAATGC-3’ | Target the 16S rRNA gene | | |
| qPCR-16S-R | 5’-CGGAAGCCACGGTTCAAGAC-3’ |  |  |  |
| qPCR-desB-F | 5’-GCGTAACTGTCTGGCGAACTC-3’ | Target the *desB* gene | | |
| qPCR-desB-R | 5’-GCGGTGCTGGCGGTTATATTG -3’ |  |  |  |
| qPCR-desC-F | 5’-TCCGCCGAACGAATCTGCTC-3’ | Target the *desC* gene | | |
| qPCR-desC-R | 5’-TGACCGCACGCAACAATCTG-3’ |  |  |  |
